# Disparity in Obesity and Hypertension Risks Observed Between Pacific Islander and Asian American Health Fair Attendees in Los Angeles, 2011–2019

**DOI:** 10.1007/s40615-022-01300-y

**Published:** 2022-04-14

**Authors:** Hong-Ho Yang, Suraj Avinash Dhanjani, Won Jong Chwa, Burton Cowgill, Gilbert Gee

**Affiliations:** 1 David Geffen School of Medicine at UCLA, Los Angeles, CA USA; 2 Johns Hopkins University School of Medicine, Baltimore, MD USA; 3 Saint Louis University School of Medicine, St. Louis, MO USA; 4 Fielding School of Public Health, Department of Health Policy and Management, University of California, Los Angeles, Los Angeles, CA USA; 5 Fielding School of Public Health, Department of Community Health Sciences, University of California, Los Angeles, 650 Charles Young Drive South, 46-081C, CHS, Los Angeles, CA 90095 USA

**Keywords:** Health disparity, Obesity, Hypertension, Pacific Islander, Asian Americans

## Abstract

**Introduction:**

The Pacific Islander American population is understudied due to being aggregated with Asian Americans. In this study, we conduct a comparative analysis of directly measured body mass index (BMI), body fat percentage (%BF), and blood pressure (BP) between Pacific Islander Americans and Asian Americans from health screenings in Los Angeles, California. We hope to reveal intra-APIA health disparities masked by this data aggregation.

**Methods:**

We analyzed BMI, %BF, and BP that were objectively measured by trained personnel at health screenings in Los Angeles between January 2011 and December 2019. We performed multivariable multinomial logistic regression models with obesity and hypertensive categories as outcome variables and ethnicity as the primary independent variable of interest. Models controlled for year of visit, participant age, sex, income, education level, years living in the USA, employment status, English proficiency, regular doctor access, and health insurance status.

**Results:**

A total of 4,832 individuals were included in the analysis. Multivariable analyses revealed that Pacific Islander participants were at significantly higher risks for being classified as obese compared with all Asian American subgroups studied, including Chinese, Korean, Thai, Vietnamese, Filipino, and Japanese. Pacific Islanders also exhibited significantly lower predicted probability of having a normal blood pressure compared with Chinese and Thai participants. Some variation between Asian subgroups were also observed.

**Conclusions:**

Pacific Islander participants had higher risk of several sentinel health problems compared to Asian American participants. Disaggregation of PI Americans from the APIA umbrella category in future studies is necessary to unmask the critical needs of this important community.

## Introduction

Asian and Pacific Islander American (APIA) is an umbrella term inclusive of heterogeneous individuals from diverse cultural and socioeconomic (SES) backgrounds [1, 2]. While significant disparities in healthcare access and health status exist within this overarching category, these communities are often aggregated in studies [2–4]. This is most apparent among Pacific Islanders (PIs), a severely understudied group who tends to perform poorly on many indicators of health outcomes compared to their Asian counterparts [5–7]. Recently, PI Americans were disproportionately affected by COVID-19, having one of the highest per-capita death rates among all ethnic groups. Asian Americans, on the other hand, had one of the lowest per-capita death rates [8]. Therefore, the aggregation of these groups can conceal the devastating impact of COVID-19 on the PI community.

Since the Office of Management and Budget’s acknowledgement of PI as its own separate racial category in 1997, there has been a growing number of studies dedicated specifically to the PI community [9]. Indeed, in 2014, the National Health Interview Survey (NHIS) launched the NHIS-Native Hawaiian and Pacific Islander (NHPI) initiative, which included a representative sample of 3,000 members of the NHPI community [10]. Several important studies from this supplement pertaining to vaccination rates, mental health, and social determinants of health have since emerged [1, 11–14]. Yet, there remains insufficient research on this growing community, which was apparent during the early phase of the COVID-19 pandemic when many states did not disaggregate or even publish data on the PI population [15, 16]. Furthermore, despite some effort to conduct disaggregated analysis, much of the scientific literature and epidemiological reports still aggregate PI under the same category as Asian Americans [17–22]. In fact, a recent report from a 3-day workshop that was sponsored by 8 NIH institutes concluded that data on the APIA population is largely limited and most existing data are not appropriately disaggregated [23].

Additional analysis comparing the health risks of PI Americans and Asian Americans is needed to further accentuate the dissimilarities between these groups. Several studies found that rates of obese body mass index (BMI) were higher among PIs compared to non-Hispanic White adults and Asians [24–28]. However, the height and weight data used to construct BMI in most of these studies were not directly measured using standard assessment procedures but reported by participants. Self-reported height and weight are less accurate than directly measured height and weight and subject to various reporting biases [29]. Therefore, measurements performed by trained personnel may provide more meaningful information pertaining health status.

Furthermore, none of these studies investigated more direct measures of body fat when determining obesity status of participants. BMI alone has been shown to be a poor measure of obesity, and the ideal cutoffs for BMI can be largely heterogeneous among different ethnic groups [30–32]. Specifically, compared to Europeans, PIs were found to have a lower body fat percentage (%BF) at the same BMI [31]. As a result, when only BMI is utilized in cross-ethnic comparisons, the extent of disparity may be inflated. In the present study, we include both BMI and %BF in our evaluation of obesity risks, in hopes of addressing these concerns and capturing a more holistic picture of health disparities between PI Americans and Asian Americans.

Hypertension is another common indicator of chronic disease risks and PI Americans were recently found to also be at a significantly higher risk for hypertension compared to the general US population [33, 34]. However, since obesity has been the primary topic of focus in the literature for PI research, hypertension has not been extensively investigated among PI Americans [24–28, 30, 35]. Since hypertension is a condition for which Asian Americans are also at higher risk for compared to the general public, it is unclear how the burden of hypertension compares between PI and Asian Americans [36].

Our study seeks to fill the gap in the literature by examining the health status of PI Americans as compared to their Asian American counterparts using a multiyear sample of adult APIA health fair participants in Los Angeles, California. Specifically, we employ directly measured BMI, %BF, and blood pressure (BP) data, which is often unavailable in larger survey datasets, to assess the differences in obesity and hypertension risks between PI American and Asian American participants while controlling for a variety of demographic and SES variables. We hypothesize that PI participants would have poorer indicators of chronic disease risks compared to participants of all Asian subgroups in our sample.

## Materials and Methods

### Data Collection

Deidentified data for this study were provided by a volunteer organization that conducts free health screenings in APIA-dense ethnic enclaves in the Greater Los Angeles area [37]. Screenings were typically conducted at community centers, churches, and cultural festivals. From 2011 to 2019, a total of 5991 participants from 88 screening events were seen. All screening activities were supervised by attending physicians and nursing faculty from the David Geffen School of Medicine at UCLA and UCLA School of Nursing. All participants provided signatures for informed, written consent. Study approval was obtained, and analysis was deemed exempt from the UCLA Institutional Review Board.

### Variables of Interest

Weight was measured with a standardized weight scale, and height was measured with a stadiometer by trained undergraduate students. BMI was calculated as kilograms/meters^2^, then classified using standard cut points for determining obesity status. The WHO has outlined specific BMI cutoffs for Asians only. Therefore, in accordance with the WHO recommendation, we adapted the following WHO Asian BMI risk cut points: < 18.5 kg/m^2^ (underweight), 18.5–22.9 kg/m^2^ (normal weight), 23–27.4 kg/m^2^ (overweight), and ≥ 27.5 kg/m^2^ (obese) [38]. For PI participants, WHO standard cutoffs were employed, which is as follows: < 18.5 kg/m^2^ (underweight), 18.5–24.9 kg/m^2^ (normal), 25–29.9 kg/m^2^ (overweight), and ≥ 30 kg/m^2^ (obese) [39].

Percent body fat (%BF) was measured by trained undergraduate students using Omron HBF-306C Handheld Body Fat Loss Monitor. Numerous studies on Chinese, Koreans, and Japanese have yielded evidence for ideal cutoffs of obese %BF to be similar to that of the general population [40–42]. Therefore, we adapted the following previously developed categorization criteria on %BF for all participants: for men, less than 20.1% was classified as healthy, 20.1–24.9% as overweight, and at or over 25% as obese; for women, less than 30.1% was classified as healthy, 30.1–34.9% as overweight, and at or over 35% as obese [43].

BP was recorded by trained medical students with participants seated and feet uncrossed using a sphygmomanometer and stethoscope. According to most recent guidelines from the American Heart Association/American College of Cardiology, values of less than 120 mmHg systolic and less than 80 mmHg diastolic were classified as normal, 120–129 mmHg systolic or less than 80 mmHg diastolic as elevated, 130–139 mmHg systolic or 80–89 mmHg diastolic as stage 1 hypertension, and at least 140 mmHg systolic or at least 90 mmHg diastolic as stage 2 hypertension. A participant who met criteria for multiple BP categories based on their systolic and diastolic readings was classified into the more pathologic category [44].

Participant demographic and SES information were self-reported on surveys. Details regarding survey questions were documented in Yang et al. [37]. This included age, sex, education, income, employment status, doctor access, health insurance status, years living in the USA, year of screening visit, ethnicity, and employment status. Ethnicity was separated into Pacific Islander, Chinese, Korean, Thai, Vietnamese, Filipino, Japanese, and other Asian ethnicities (detailed breakdown in Table 1). Ideally, the Pacific Islander subgroups (e.g., Samoan, Tongan) would be studied, but they were not asked in the survey which was initially designed by the service organization.

**Table 1:** Participant Demographic and Socioeconomic Status Profile, 2011–2019 (*N* = 4832)

Covariate	Sub-category	Percent (%)
Ethnicity	Chinese	51.6
	Korean	19.0
	Thai	14.8
	Vietnamese	6.3
	Filipino	2.1
	Pacific Islander	1.9
	Japanese	1.5
	Other Asian	2.8 (*n* = 137)^+^
Age	18–40	17.7
	40–65	60.7
	65 +	21.6
Sex	Female	61.8
	Male	38.2
Income	< 20 k	57.8
	20–40 k	21.3
	40–60 k	10.9
	≥ 60 k	10.0
Education Level	Less than high school	14.0
	High school	28.7
	College or higher	57.4
Years in US	0–5	15.9
	5–10	8.8
	10–15	16.5
	15 +	58.9
Employment	Not working	25.1
	Part-time	18.4
	Full-time	30.8
	Retired	25.7
English Proficiency	Low english proficiency	47.8
	High english proficiency	52.2
Have Health Insurance	Yes	54.8
Have Doctor	Yes	46.9
Last Doctor Visit	This month	13.4
	This year	28.3
	1 + year ago	21.5
	2 + years ago	12.7
	Don’t remember	24.1

^+^Other Asian includes Bengali (1), Bangladesh (3), Burmese (1), Cambodian (15), Hmong (1), Indian (20), Indonesian (52), Khmer (1), Laos (2), Malaysian (5), Mien (1), Mongolian (3), Pakistan (1), Singaporean (1), Sri Lankan (18), and not specified (12)

### Exclusion Criteria and Data Imputation

Initially, our sample included 5991 observations. We excluded Hispanic (*n* = 347), White (*n* = 167), Black (*n* = 46), and other non-Asian (*n* = 104) participants due to our interest in studying Asians and PIs. Further, 407 persons with missing ethnicity information and 88 duplicated responses from participants who attended more than one screening were excluded, leaving 4832 observations for analysis. Different exclusion criteria were applied for each outcome variable model. For the BMI model, 241 observations with missing BMI measurements and 170 observations in the underweight category excluded (no PI or Filipino participant was in the underweight range), leaving *n* = 4421 for analysis. For the %BF model, 730 observations with missing %BF measurements were excluded, leaving *n* = 4102 for analysis. Finally, for the BP model, 588 observations with missing BP measurements were excluded, leaving *n* = 4244 for analysis.

Missing data for variables other than ethnicity, BMI, %BF, and BP were addressed by multiple imputation and the Markov Chain Monte Carlo Method as recommended by previous literature to optimize stability of imputed variables [45].

### Statistical Analysis

Three separate multivariable models were constructed for the three outcome variables of interest: BMI, %BF, and BP categories. Since all three outcome variables were ordinal, parallel line fit tests were performed for model selection [46]. Results indicated that parallel regression assumption was not met for all models. Therefore, multinomial regression was implemented. In each model, year of visit, participant age, sex, income, education level, years living in the USA, employment status, English proficiency, regular doctor access, and health insurance status were incorporated as covariates. All statistical analyses were conducted through Stata software, Version 13.

## Results

A summary of demographic and socioeconomic profile of study participants is provided in Table 1. A total of 4832 participant responses from 2011 to 2019 were included in the analysis. Our sample heavily consisted of participants of Chinese descent (51.6%), followed by Korean (19.0%) and Thai (14.8%). PI made up 1.9% of our sample. Most participants were between the ages of 40 and 65 (60.7%), females (61.8%), earned an income of less than $20,000 (57.8%), had an education level of college or higher (57.4%), and had been in the USA for longer than 15 years (58.9%). There was a roughly even split between participants reporting an employment status of “not working,” “part-time,” “full-time,” and “retired” (25.1%, 18.4%, 30.8%, and 25.7%, respectively), and participants reporting high English proficiency and low English proficiency in our sample (52.2% vs. 47.8%, respectively).

Healthcare access variables also evenly divided our sample, with 54.8% of our participants indicating having a health insurance plan and 46.9% of our participants indicating having a regular physician. Almost half of our participants reported being last seen by a physician within a year (41.7%), 21.5% of participants reported being last seen by a physician more than one year ago, and 12.7% of participants reported being last seen by a physician more than two years ago at the time of encounter. The remaining participants indicated that they did not remember.

A summary of participant raw %BF, BMI, and BP categories stratified by ethnicity is presented in Table 2. Around 56% of PIs had a %BF in the obese range, compared to 40 to 49% among Asian subgroups. Around 67% of PIs had a BMI in the obese range, compared to 12 to 30% among Asian subgroups. Finally, 36.9% of PIs had a BP reading in the stage 1 hypertension range and 21.4% of PIs had a BP reading in the stage 2 hypertension range, compared to an average of 32.0% for stage 1 hypertension and an average of 25.8% for stage 2 hypertension among Asian ethnic subgroups.

**Table 2:** Participant percent body fat, body mass index, and blood pressure categories stratified by ethnicity, Los Angeles, California, 2011–2019 (*N* = 4832)

Category	Pacific Islander	Chinese	Korean	Thai	Vietnamese	Filipino	Japanese	Other Asian	Total
Percent body fat (%BF)									
Normal	16.3%	31.0%	29.1%	29.4%	36.1%	30.4%	35.6%	27.1%	30.4%
Overweight	27.5%	27.7%	30.4%	27.1%	24.2%	22.8%	20.3%	24.3%	27.7%
Obese	56.3%	41.2%	40.5%	43.5%	39.8%	46.8%	44.1%	48.6%	42.0%
Body mass index (BMI)									
Underweight	0.0%	4.2%	3.2%	3.4%	4.5%	0.0%	5.8%	2.3%	3.7%
Normal	13.8%	37.4%	35.4%	35.9%	33.0%	51.1%	46.4%	30.3%	36.3%
Overweight	19.5%	43.7%	49.3%	42.9%	48.3%	35.2%	34.8%	37.9%	44.0%
Obese	66.7%	14.7%	12.1%	17.8%	14.2%	13.6%	13.0%	29.5%	16.0%
Blood pressure (BP)									
Normal	32.1%	32.2%	23.8%	35.1%	28.0%	27.0%	25.8%	30.8%	30.5%
Elevated	9.5%	12.5%	11.8%	10.4%	11.3%	12.4%	22.6%	11.7%	12.1%
Stage 1 hypertension	36.9%	30.0%	33.2%	31.8%	35.6%	30.3%	29.0%	34.2%	31.4%
Stage 2 hypertension	21.4%	25.3%	31.2%	22.7%	25.1%	30.3%	22.6%	23.3%	26.0%

### Body Mass Index

**Fig. 1:**
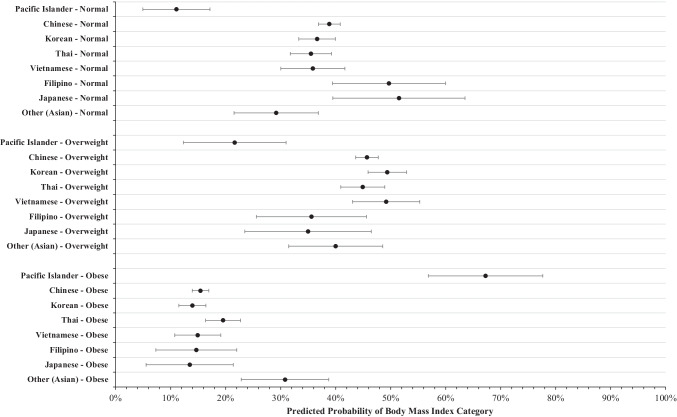
Predicted probability of body mass index categories between ethnic subgroups. Marginal predicted probability and 95% confidence intervals were computed from a multinomial logistic regression model with body mass index as the nominal outcome variable controlling for year of visit, participant age, sex, income, education level, years living in the USA, employment status, English proficiency, regular doctor access, and health insurance status

Results from the multivariable model with BMI category as the outcome variable are illustrated in Fig. 1. Analysis revealed that with all other covariates held at the mean, the predicted probability of PIs having a BMI in the normal category is 11.1% (95% C.I. = [5.0, 17.2]), which is significantly lower than that of all Asian ethnic groups studied, including Chinese (95% C.I. = [36.9, 40.8]), Korean (95% C.I. = [33.3, 40.0]), Thai (95% C.I. = [31.8, 39.3]), Vietnamese (95% C.I. = [30.1, 41.7]), Filipino (95% C.I. = [39.4, 60.0]), and Japanese (95% C.I. = [39.5, 63.5]).

The predicted probability of PIs having a BMI in the overweight category is 21.7% (95% C.I. = [12.3, 31.0]), which is significantly lower than that of Chinese (95% C.I. = [43.6, 47.8]), Korean (95% C.I. = [45.9, 52.9]), Thai (95% C.I. = [40.9, 48.9]), and Vietnamese (95% C.I. = [43.1, 55.3]).

Finally, the predicted probability of PIs having an obese BMI reading is 67.3% (95% C.I. = [56.9, 77.7]), which is significantly higher than that of all Asian ethnic groups studied, including Chinese (95% C.I. = [13.9, 16.9]), Korean (95% C.I. = [11.5, 16.4]), Thai (95% C.I. = [16.4, 22.7]), Vietnamese (95% C.I. = [10.7, 19.1]), Filipino (95% C.I. = [7.3, 22.0]), and Japanese (95% C.I. = [5.6, 21.4]).

### Body Fat Percentage

**Fig. 2:**
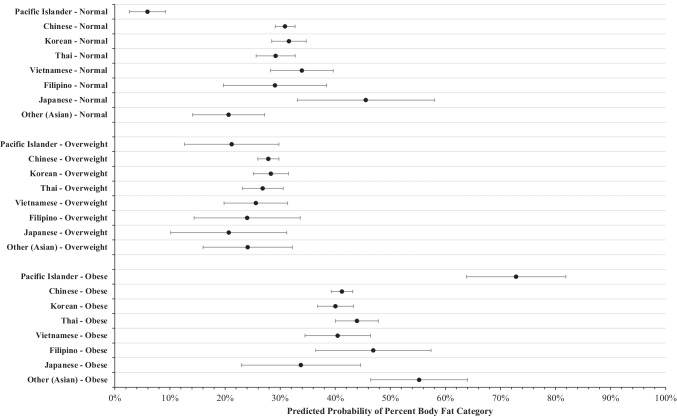
Predicted probability percent body fat categories between ethnic subgroups. Marginal predicted probability and 95% confidence intervals were computed from a multinomial logistic regression model with body fat percentage as the nominal outcome variable controlling for year of visit, participant age, sex, income, education level, years living in the USA, employment status, English proficiency, regular doctor access, and health insurance status

Results from the multivariable model with %BF category as the outcome variable are illustrated in Fig. 2. Analysis revealed that with all other covariates held at the mean, the predicted probability of PIs having a %BF in the normal category is 5.9% (95% C.I. = [2.7, 9.2]), which is significantly lower than that of all Asian ethnic groups studied, including Chinese (95% C.I. = [29.1, 32.7]), Korean (95% C.I. = [28.5, 34.7]), Thai (95% C.I. = [25.6, 32.8]), Vietnamese (95% C.I. = [28.3, 39.6]), Filipino (95% C.I. = [19.7, 38.4]), and Japanese (95% C.I. = [33.1, 58.0]). However, the predicted probability of PIs having an overweight %BF did not differ significantly from any Asian subgroups studied.

Finally, the predicted probability of PIs having an obese %BF is 72.9% (95% C.I. = [63.8, 81.9]), which is significantly higher than all Asian subgroups studied, including Chinese (95% C.I. = [39.3, 43.2]), Korean (95% C.I. = [36.8, 43.3]), Thai (95% C.I. = [40.7, 41.8]), Vietnamese (95% C.I. = [34.5, 46.4]), Filipino (95% C.I. = [36.4, 57.4]), and Japanese (95% C.I. = [22.9, 44.6]).

### Blood Pressure

**Fig. 3:**
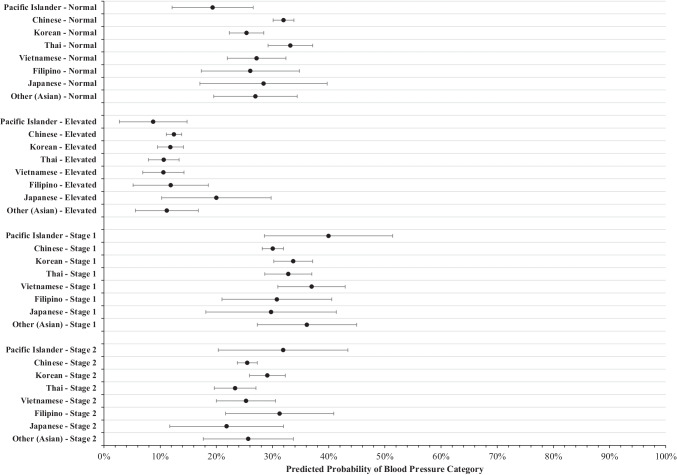
Predicted probability of blood pressure categories between ethnic subgroups. Marginal predicted probability and 95% confidence intervals were computed from a multinomial logistic regression model with blood pressure as the outcome variable controlling for year of visit, participant age, sex, income, education level, years living in the USA, employment status, English proficiency, regular doctor access, and health insurance status

Results from the multivariable model with BP category as the outcome variable are illustrated in Fig. 3. With all other covariates held at the mean, the predicted probability of PIs having a normal BP is 19.3% (95% C.I. = [12.1, 26.6]), which is significantly lower than that of Chinese (95% C.I. = [30.1, 33.8]) and Thai (95% C.I. = [29.2, 37.2]).

## Discussion

Our research shows that Pacific Islander health fair participants exhibited significantly higher risks of being classified as obese compared with all subgroups of Asian American health fair participants. Pacific Islander participants were also less likely to have a normal blood pressure compared with Chinese and Thai participants.

### Disparity in Obesity Rates Between Asian and PI American Health Fair Participants

Previous studies have consistently identified higher risks of unhealthy BMI among PI Americans compared to Asians Americans [25–28]. Our research, using objectively measured indicators, corroborates past research that was based on self-reported data. In our sample, the odds of obesity among PIs was significantly higher than that of all Asian American subgroups. A previous study found that a higher percentage of Native Hawaiian and PI ancestry within participants, as opposed to Asian ancestry, was significantly correlated with an increased BMI, and that the higher BMI was accounted for by differences in educational attainment [28]. Our analyses suggest that the higher rates persist even after accounting for education, as well as other indicators of social status, including income, employment, and health insurance. We speculate that a key difference in the results lie in the setting, whereby accounting for education in Hawaii provides a qualitatively greater equalization in social status for Native Hawaiian and PI communities compared to California. Further emphasis on the disaggregation of PI Americans from the broad APIA category in reports is needed to unmask the specific health risks PI Americans face.

In addition to BMI, %BF is also an important indicator of obesity. However, comparative studies of %BF between PI Americans and Asian Americans are very limited in the literature and many previous studies employed only BMI and waist circumference as the sole indicators of obesity [25–28]. One of the very few studies that assessed %BF among PIs was a small-scale investigation involving 55 young PI women that measured %BF employing X-ray absorptiometry equipment and found a positive association between proportion of PI ancestry and %BF [35]. Our finding, with more diverse participants, adds to this limited body of literature by confirming that PI Americans are at a significantly higher risk for having an obese %BF compared to all Asian American subgroups studied.

Several factors may explain the disproportionately high rates of obesity among PI participants in our sample. Prior research has proposed that nutritional transition in Pacific Islands due to importation of packaged food and adaptation of western diets, along with poor education on diet change, could elevate obesity risks [47]. This trend was similarly observed among PI Americans, as studies have identified high rates of unhealthy dietary habits, sedentary lifestyles, and insufficient physical activity among PIs [48, 49]. A recent systematic review also delineated several important prenatal, contextual, and behavioral factors that can serve as contributors of obesity among PIs [50]. Specifically, the PI population was found to practice breastfeeding, which was previously shown to be protective of obesity, significantly less than other racial groups [51]. Poor neighborhood and maternal education level were also proposed as potential mediators of high obesity risks among PIs [52]. Further, transnational agreements, policies, and practices (e.g., military occupation of Island nations by the USA) may further shape health risk factors [53]. Although several compelling hypotheses were proposed, the underlying factors driving the observed high obesity rates among PIs have yet to be elucidated. Additional studies on this issue are warranted.

### Disparity in Hypertension Rates Between Asian and PI American Health Fair Participants

Blood pressure is also an understudied measure of health status in the field of disaggregated APIA research. A recent study conducted among a population of health screening participants in the US found that hypertension rates among the PI American community was higher than the national average across all racial groups [33]. While this study did not directly compare PI Americans’ rates of hypertension with their Asian American counterparts, the finding suggests that PI Americans experience a disproportionate burden of hypertension. Similarly, we also observed lower rates of normal blood pressure among PI Americans compared to Chinese and Thai. However, the disparity in hypertension between PI and Asians in our study was not as pronounced as the disparity we observed in obesity. This is expected, considering that hypertension is a more prevalent condition among the Asian American community than obesity [36]. Nevertheless, our analysis demonstrates the importance of disaggregation even among Asian subgroups as hypertension risks of PIs were only found to be significantly different than that of Thai and Chinese, but not other Asian American subgroups we studied.

### Data Disaggregation of Asian and Pacific Islander Americans

While recent recognitions of the PI population as its own distinct group have prompted increasing literature focusing specifically on the experiences of the PI community, additional efforts to disaggregate PIs from the greater APIA umbrella category in important risk assessment studies are still warranted [11–13, 17–22]. A key reason to disaggregate is to uncover communities that are at particularly high risk for a certain condition, and also, to better understand the context of risk factors for a given community such as immigration patterns, dietary practices, and educational levels. As our analyses showed, the health profiles for Asians and PIs differ significantly, and assumptions that these communities are similar with regard to health risk factors such as diet and education are unwarranted. Furthermore, as PI communities tend to be numerically smaller, the potential that their needs are unrecognized and unmet are greater than that of Asian Americans. For example, important health education materials might be translated into Chinese, but not translated into Tongan.

Future research should also consider circumstances when aggregation might be appropriate. As noted in a previous study, disaggregation need not always be based on ethnic subgroup [54]. In some contexts, it may be useful to group individuals based on refugee status rather than on ethnicity. There may even be circumstances where aggregation is appropriate, such as when the hypothesis that hate crimes are perpetrated equally against all Asians and Pacific Islanders due to that the “they all look alike” phenomenon is investigated [54]. Therefore, disaggregation of data is desirable and warranted, but the reasons for disaggregation should always be made clear in research.

### Limitations

While our study provides valuable information regarding the health status of PI Americans, it is not without its limitations. First, it is important to note that data for this study were collected among community health fair participants in APIA-dense areas. Although our dataset provides directly and objectively measured BMI, %BF, and blood pressure that are often unavailable in larger probability samples, individuals in this cohort may in some way differ from the general APIA population. For instance, since they seek free medical services, they could be less likely to have health insurance and a regular source of care. Our APIA sample is also overrepresented by Chinese, middle-aged, and low socioeconomic status individuals. Therefore, our findings are most appropriately generalized to APIAs resembling the characteristics mentioned above. Nevertheless, considering the limited data on objectively measured indicators of obesity and hypertension among PI Americans, our findings can contribute significantly to the literature.

Furthermore, the proportion of PI participants is small compared to Asian participants in our sample (1.9% vs. 98.1%). However, this ratio (1:52) is relatively similar to the demographic distribution of Los Angeles County, with PI Americans making up 0.3% versus Asian Americans making up 13.8% of the population in 2011 (1:44) and PI Americans making up 0.3% versus Asian Americans making up 14.7% of the population in 2019 (1:49) [55]. Therefore, our comparison is reflective of the proportions of these populations in Los Angeles.

Finally, we are unable to disaggregate among the Pacific Islander subgroups. Ideally, we would be able to distinguish between Native Hawaiians, Samoans, Tongans, and other important groups. This research provides important novel information for the PI community, but future research would collect larger samples of these subgroups and provide disaggregated analyses.Despite these limitations, our study addresses a critically understudied topic in the literature. We provide a diverse APIA cohort and a multiyear dataset with consistent, objective measurements of obesity and hypertension by trained students under supervision of physicians. To our knowledge, this is the first study to evaluate BMI, %BF, and BP together with consistent standard when examining disparity in health status between PI and Asian Americans. We believe our study will be a significant addition to the literature and raise an important issue for the public health community.

## Conclusion

Upon conducting disaggregated analysis of BMI, %BF, and BP among a sample of APIA health fair attendees, we found that obesity and hypertension risks were consistently higher among PI Americans compared to Asian Americans. Yet, when these groups are aggregated, the concerning health disparities would not have been uncovered. Therefore, this arbitrary grouping may be harmful by masking the critical needs of PI Americans. Intentional efforts in future studies to disaggregate PI Americans from the APIA umbrella category are necessary to address the obstacles PI Americans face and promote the much-needed public health attention they deserve.
